# Fibroblast growth factor 2 inhibits the expression of stromal cell-derived factor 1α in periodontal ligament cells derived from human permanent teeth *in vitro*

**DOI:** 10.3892/ijmm.2011.869

**Published:** 2011-12-23

**Authors:** TAKEYOSHI ASAKAWA, NAOYUKI CHOSA, YOSHITAKA YOSHIMURA, ASAMI ASAKAWA, MITSURO TANAKA, AKIRA ISHISAKI, MASATO MITOME, TOMOKAZU HASEGAWA

**Affiliations:** 1 Department of Pediatric Dentistry, School of Dentistry, Iwate Medical University, Morioka 020-8505; 2 Department of Oral Biochemistry, School of Dentistry, Iwate Medical University, Morioka 020-8505; 3 Department of Molecular Cell Pharmacology, Division of Oral Pathological Science, Hokkaido University Graduate School of Dental Medicine, Kita-ku, Sapporo 060-8586; 4 Department of Pediatric Dentistry, Institute of Health Biosciences, University of Tokushima Graduate School, Tokushima 770-8504; 5 Department of Pediatric Dentistry, Tokushima University Hospital, Tokushima 770-8504, Japan

**Keywords:** stromal cell-derived factor 1α, fibroblast growth factor 2, periodontal ligament cells, permanent tooth

## Abstract

Although cells derived from periodontal ligament (PDL) tissue are reported to have stem cell-like activity and are speculated to play a crucial role for tissue healing and regeneration after injury or orthodontic treatment, mechanisms regulating their recruitment and activation remain unknown. Recently, stromal cell-derived factor 1α (SDF-1α) has been reported to be important for stem cell homing and recruitment to injured sites. The aim of this study was to evaluate whether fibroblast growth factor 2 (FGF-2) affects the expression of SDF-1α in PDL cells derived from human permanent teeth *in vitro*. Using real-time PCR, the expression of SDF-1α mRNA in PDL cells was inhibited by treatment with 10 ng/ml FGF-2. When PDL cells were treated with SU5402 (an inhibitor of FGF receptor 1) in combination with FGF-2, the FGF-2-reduced expression of SDF-1α was inhibited. In the presence of the JNK inhibitor SP600125, SDF-1α mRNA in PDL cells was not suppressed by the FGF-2 treatment. Western blot analysis also showed that SDF-1α production was suppressed by treatment with FGF-2, but it recovered with treatment by FGF-2 + SU5402. These findings suggest that SDF-1α from PDL cells plays an important role in the regeneration and homeostasis of periodontal tissues via the recruitment of stem cells.

## Introduction

The periodontal ligament (PDL) is located between the tooth root and alveolar bone (1). Most PDL cells are fibroblasts with relatively high alkaline phosphatase (ALP) activity (2,3). Fibroblasts derived from the PDL have the ability to form bone-like tissues *in vitro*, similar to osteoblasts (2,3), and thus, PDL cells function similarly to osteoblasts in hard tissue formation. Recently, several studies have demonstrated that PDL cells also differentiate into cementoblastic and adipogenic cells *in vitro* (4). Therefore, the PDL probably contains pluripotent progenitor cells or putative stem cells. However, the mechanism of PDL cell migration is poorly understood.

Stromal cell-derived factor 1α (SDF-1α, also known as CXCL12) is an α-chemokine that strongly chemoattracts mesenchymal stem cells (MSCs) and endothelial progenitor cells (EPCs) via interaction with its unique receptor CXCR4 (5). In adults, tissue repair and regeneration after injury are thought to involve the selective recruitment of circulating or resident stem cell populations. The importance of SDF-1α in stem and progenitor cell recruitment has been established by showing that its expression in injured tissue correlates with the recruitment of adult stem cells and tissue regeneration (5–8). Therefore, SDF-1α, as a type of stem cell-development factor and chemokine, plays an important role in coordinating tissue injury and repair.

For regenerative therapy, biologically active soluble factors such as cytokines and growth factors are being evaluated for clinical use in the regeneration of periodontal tissue damaged or lost as a result of periodontitis. Of these factors, fibroblast growth factor 2 (FGF-2) is a multifunctional growth factor that has a variety of effects, including the induction of proliferation and differentiation in a wide range of mesodermal and neuro-ectodermal cells (9). Therefore, we investigated whether FGF-2 could regulate the expression of SDF-1α in cultured PDL cells *in vitro*.

## Materials and methods

### Reagents

FGF-2 was obtained from R&D Systems (Minneapolis, MN, USA). Anti-SDF-1α polyclonal antibody for the western blot analysis was obtained from Abcam (ab9797, Cambridge, UK). SU5402 (10 μM), SP600125 (10 μM), U0126 (10 μM), SB203580 (10 μM) and LY294002 (10 μM) were purchased from EMD Chemicals, Inc. (Calbiochem; Gibbstown, NJ, USA).

### Cell culture

PDL tissues were obtained from the middle third of the root surfaces of healthy human permanent teeth (3 donors, aged 7–8 years), as previously described (10,11). Informed consent was obtained from the donors' parents before tooth extraction, which was carried out in our hospital during the course of orthodontic treatment. This study protocol was approved by the Ethics Committee of the Iwate Medical University, School of Dentistry (no. 01101).

The PDL tissues were cut into pieces using a surgical blade and were digested with collagenase (2 mg/ml) at 37˚C for 30 min. The tissues were then washed with Dulbecco's phosphate-buffered saline (PBS), placed on culture dishes, and maintained in α-modified minimum essential medium (α-MEM; Life Technologies Corp., Carlsbad, CA, USA) supplemented with 10% fetal bovine serum (FBS; Life Technologies Corp.). Fibroblastic cells that grew out from the PDL tissues were used as PDL cells. When the cells reached confluence, they were detached with 0.2% trypsin and 0.02% EDTA·4Na in PBS and subcultured at a 1:4 split ratio. All of the experiments were performed using fourth passage cells cultured in α-MEM supplemented with 10% FBS in the absence or presence of 10 ng/ml FGF-2 for between 24 and 48 h. All of the cultures were maintained at 37˚C in a humidified atmosphere of 5% CO_2_ in air.

### Isolation of total-RNA

Total-RNA was extracted from the cultured PDL cells using ISOGEN (Nippon Gene, Tokyo, Japan) as described previously (10,11). The pellet of total-RNA was washed briefly with 75% ethanol, resuspended in 30 μl of diethylpyrocarbonate (DEPC)-treated water, and stored at −80˚C. The concentration of total-RNA was determined spectrophotometrically by measuring the optical density at 260 nm.

### Quantitative real-time reverse transcription-polymerase chain reaction

One microgram of the RNA sample was reverse-transcribed to first-strand cDNA using a PrimeScript RT reagent kit (Takara Bio, Inc., Shiga, Japan) according to the manufacturer's protocol. A Thermal Cycler Dice real-time system (Takara Bio, Inc.) was used for the two-step reverse transcription-polymerase chain reaction. The cDNA was amplified with SYBR Premix ExTaq (Takara Bio, Inc.) and specific oligonucleotide primers for target sequences encoding parts of SDF-1α. The primers (Table I) were designed based on the cDNA sequences of human mRNA for SDF-1α and glyceraldehyde-3-phosphate dehydrogenase (GAPDH). Amplification conditions consisted of 10 sec at 95˚C, followed by 40 cycles at 95˚C for 5 sec and 60˚C for 30 sec, with a final 15 sec at 95˚C and 30 sec at 60˚C in the Thermal Cycler Dice real-time system (12,13).

### Western blot analysis of SDF-1α expression from PDL cells

After treatment with FGF-2 for 7 days, the conditioned media from PDL cell culture were collected for SDF-1α analysis. Subsequently, 20 μl of conditioned media were dissolved in SDS buffer without dithiothreitol, incubated at 95˚C for 5 min, resolved electrophoretically on 10% SDS-polyacrylamide gels and transferred to a polyvinylidene difluoride membrane (Millipore Corp., Bedford, MA, USA). After being blocked with 5% skim milk in Tris-buffered saline containing 0.1% Tween-20 (TBST), the membrane was incubated with mouse anti-human SDF-1α antibodies and subsequently with anti-mouse secondary antibodies (Life Technologies Corp.). Specific protein bands on the membrane were detected using an enhanced AP conjugate substrate kit (Bio-Rad Laboratories, Inc., Hercules, CA, USA) as previously described (10–13).

### Statistical analysis

The results are expressed as means ± SEM. Statistical significance was determined using one-way analysis of variance with Bonferroni post hoc comparisons between pairs of groups. The threshold for statistical significance was set *a priori* at P<0.01.

## Results

### FGF-2 induces morphological changes

**Figure 1:**
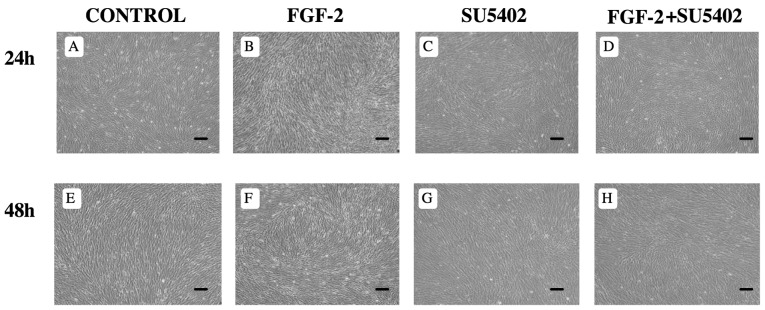
Effect of FGF-2 on the morphology of PDL cells derived from human permanent teeth. PDL cells cultured in control medium for 24 h (A) and 48 h (E) showed confluence. PDL cells cultured in the presence of SU5402 (C and G) showed no differences from PDL cells cultured in control medium. When PDL cells were cultured in the presence of FGF-2 for 24 h (B) and 48 h (F), the PDL cell morphology was altered into long, thin, spindle-shaped fibroblastic cells. PDL cells cultured in both FGF-2 and SU5402 (FGF-2 + SU5402) were similar to those cultured in control medium (D and H). Bar, 100 μm.

Morphological changes in PDL cells were induced by treatment with FGF-2 for 24–48 h. After culturing for 24–48 h, PDL cells reached confluence in control media (Fig. 1A and E) and in the presence of FGF-2 (Fig. 1B and F). When treated with FGF-2, PDL cells altered their morphology into long, thin, spindle-shaped fibroblasts (Fig. 1B and F). There were no differences in the appearance of PDL cells between control and SU5402 treatment (Fig. 1A, C, E and G), or between control and FGF-2 + SU5402 (Fig. 1A, C, D and H).

### FGF-2 suppresses SDF-1α mRNA expression

**Figure 2:**
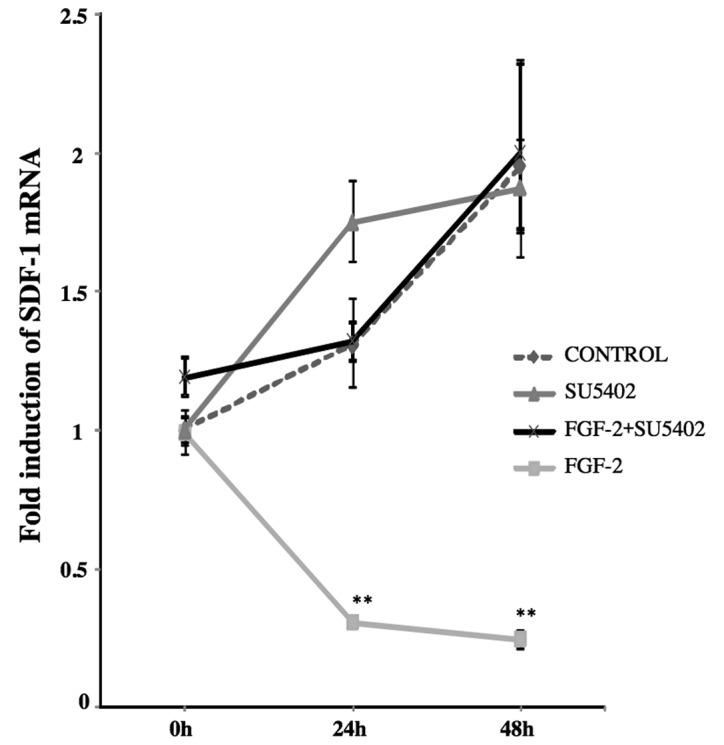
Real-time PCR analysis for SDF-1α mRNA expression. The expression of SDF-1α mRNA in PDL cells increased at 24 and 48 h when cultured in the control media and in the presence of SU5402 and FGF-2 + SU5402. Expression of SDF-1α mRNA in PDL cells treated with FGF-2 was significantly reduced at 24 and 48 h. Values are expressed as a ratio with respect to SDF-1α expression at 0 h. ^**^P<0.01.

Expression of SDF-1α mRNA was suppressed in PDL cells cultured in the presence of FGF-2 for 24 and 48 h. When PDL cells were cultured in the presence of FGF-2 for 24 and 48 h, SDF-1α mRNA expression was significantly decreased compared to the 0 h level (0 h, 1; 24 h, 0.3; 48 h, 0.2; P<0.01). However, after treating with SU5402 alone and FGF-2 + SU5402, SDF-1α expression was slightly increased (Fig. 2).

### FGF-2 decreased SDF-1α expression

**Figure 3:**
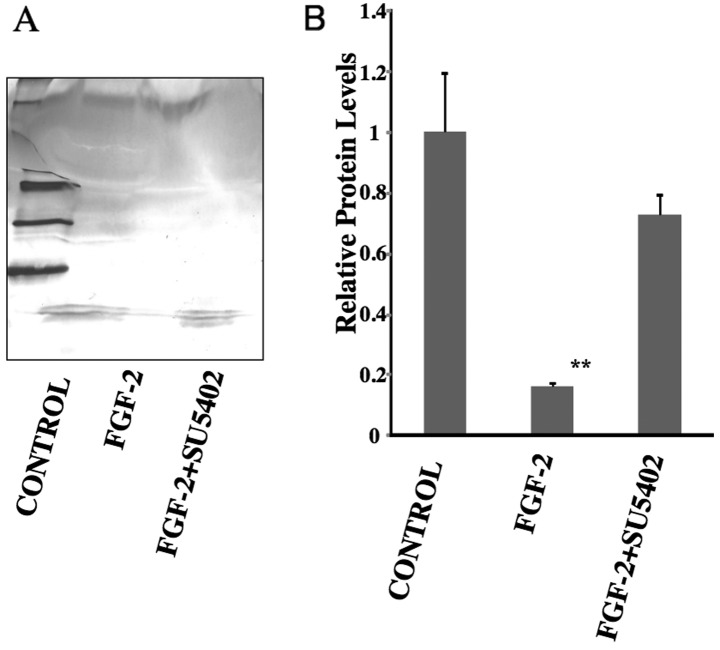
Western blot analysis of SDF-1α expression in PDL cells. (A) SDF-1α was detected in conditioned media when PDL cells were cultured in control media and in FGF-2 + SU5402 media. SDF-1α expression was reduced in the presence of FGF-2. (B) When measured with densitometry, SDF-1α expression in FGF-2-treated PDL cells was significantly decreased compared to both the control and FGF-2 + SU5402 conditions. Values are expressed as a ratio with respect to SDF-1α expression in control media. ^**^P<0.01.

SDF-1α expression decreased in PDL cells cultured in the presence of FGF-2. After treatment with FGF-2 for 7 days, the production of SDF-1α was noticeably decreased in PDL cells compared to the control (control, 1; FGF-2, 0.16; P<0.01) (Fig. 3). However, in the presence of FGF-2 + SU5402, SDF-1α expression was slightly decreased compared with control, although this was not statistically significant (control, 1; FGF-2 + SU5402, 0.72).

### SP600125 inhibites the FGF-2-mediated decrease in SDF-1α expression

**Figure 4:**
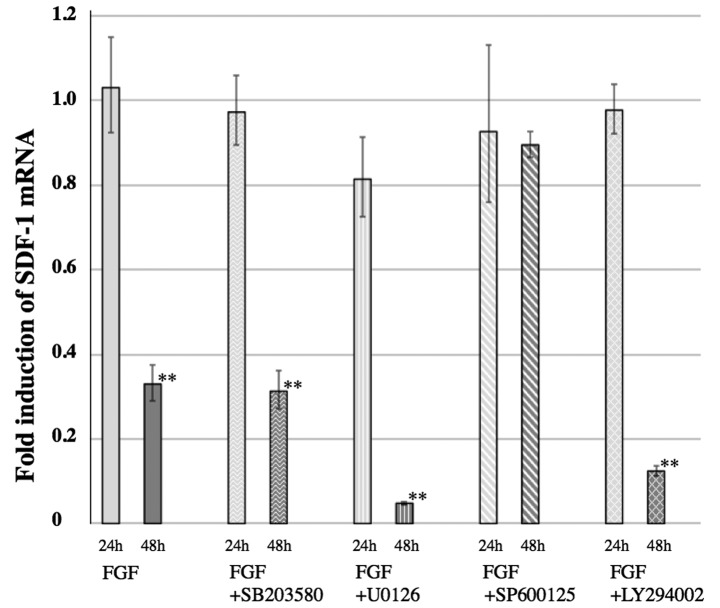
The effect of MAP kinase and Akt pathway inhibitors on SDF-1α expression. When PDL cells were cultured in the presence of various key signal pathway inhibitors (10 μmol/l each of U0126, SB203580, SP600125 and LY294002) for 48 h, SDF-1α expression was examined. The reduction of SDF-1α expression by FGF-2 treatment was inhibited by culturing PDL cells in the presence of FGF-2 + SP600125. Values are expressed as a ratio with respect to SDF-1α expression in control media. ^**^P<0.01.

The decreased expression of SDF-1α in PDL cells, mediated by FGF-2, was inhibited by SP600125, an inhibitor of JNK. Only the treatment of FGF-2 + SP600125 inhibited the decreased expression of SDF-1α observed when PDL cells were cultured with FGF-2 alone (Fig. 4). Other MAP kinase inhibitors, U0126 (an MEK1/2 inhibitor) and SB203580 (a p38 kinase inhibitor), had no effect on the decreased expression of SDF-1α. LY294002, a phosphatidylinositol-3 kinase (PI3K) inhibitor, also had no effect on the decreased expression of SDF-1α (Fig. 4).

## Discussion

This study demonstrated that SDF-1α mRNA was inhibited in cultured PDL cells by treatment with FGF-2. Decreased expression of SDF-1α was also demonstrated in conditioned media from PDL cells cultured with FGF-2. This is the first report of altered SDF-1α expression regulated by FGF-2 treatment in PDL cells derived from human permanent teeth.

PDL tissue regeneration and homeostasis in response to pathological and environmental changes, such as injury and orthodontic treatment, are thought to depend on recruitment of circulating or resident stem cells. SDF-1α plays an important role in tissue healing by recruiting endothelial progenitor cells and MSCs from the bone marrow through its receptor, CXCR4 (5–8). However, it remains unclear whether the recruitment of stem and progenitor cells can be regulated in PDL tissue.

In this study, PDL cells derived from human permanent teeth were used to investigate the effects of FGF-2 on the expression of SDF-1α, using real-time PCR. Previous studies have shown that PDL cells not stimulated with FGF-2 basally express SDF-1α (14,15). The results of the current study support those findings; PDL cells expressed SDF-1α in the absence of FGF-2 (Fig. 2). Surprisingly, PDL cells significantly decreased the expression of SDF-1α when cultured in the presence of FGF-2 (Fig. 2). In addition, this study demonstrated that SDF-1α expression was regulated by FGF-2 via the fibroblast growth factor receptor (FGFR). Results showed levels of SDF-1α expression similar to those of the control when cultured in media with combined FGF-2 and an FGF receptor antagonist, SU5402. No statistical differences in SDF-1α expression were found between untreated cells and cells treated with SU5402 alone.

Western blot analysis showed that SDF-1α protein production in conditioned media was significantly decreased by treatment with FGF-2 compared to the control group. In the presence of FGF-2 + SU5402, SDF-1α expression recovered almost to control levels (Fig. 3). Similarly to the real-time PCR results, this analysis suggests that FGF-2 inhibits SDF-1α protein expression in PDL cells via the FGFR.

A phosphatidylinositol-3 kinase (PI3K) inhibitor, LY2940002, blocks PI3K and results in the inhibition of Akt pathway activity. It has previously been shown that FGF-2-induced Akt phosphorylation depends upon PI3K in MSCs (16). In the presence of FGF-2 + LY2940002, FGF-2-reduced expression of SDF-1α was not inhibited in PDL cell culture. Therefore, the current results indicate that the PI3K/Akt pathway is not related to FGF-2-reduced expression of SDF-1α in PDL cells.

Furthermore, the specific MEK inhibitor U0126 or the p38 MAP kinase inhibitor SB202190 in combination with FGF-2 had no effect on FGF-2-induced SDF-1α inhibition (Fig. 4). However, the JNK inhibitor SP600125 in combination with FGF-2 inhibited FGF-2-reduced expression of SDF-1α (Fig. 4). These results indicate that the JNK pathway plays an important role in FGF-2-mediated effects in PDL cells.

FGF-2 has been described as a multipotent cytokine that regulates cell proliferation as well as differentiation, matrix composition, and migration in a number of cell types (9). It has also been shown that FGF-2 itself as well as SDF-1α could control MSC migration (5–8,17). SDF-1α expression levels in clinically inflamed dental pulp tissues were higher than those in healthy dental pulp (18). Moreover, human gingival fibroblasts constitutively expressed SDF-1α (19). This expression was enhanced by stimulation with tumor necrosis factor-α (TNF-α) and transforming growth factor-β (TGF-β) (19). Together with these results, it can be concluded that PDL cells in an environment where FGF-2 is abundant may decrease SDF-1α expression because FGF-2 is capable of inducing the migration of MSCs.

This is the first study to demonstrate that the treatment of PDL cells derived from human permanent teeth with FGF-2 *in vitro* regulates SDF-1α expression. These findings can aid in understanding of mechanisms of PDL tissue regeneration by suggesting that with effective regulation of SDF-1α expression, MSC migration can be controlled.

## Figures and Tables

**Table I:** Primers used in the quantitative real-time PCR reverse transcription-polymerase chain reaction (real-time PCR).

Gene name	Origonucleotide sequence (5′-3′)
SDF-1	F: GAGCCAACGTCAAGCATCTCAA R: TTTAGCTTCGGGTCAATGCACA
GAPDH	F: GCACCGTCAAGGCTGAGAAC R: TGGTGAAGACGCCAGTGGA

SDF-1, stromal cell-derived factor 1α; GAPDH, glyceraldehyde 3-phosphate dehydrogenase.
